# Coincidence or connection? A patient with concurrent Lane Hamilton Syndrome and idiopathic membranous nephropathy

**DOI:** 10.1016/j.rmcr.2021.101446

**Published:** 2021-06-07

**Authors:** Scarlett Austin, Dale Kobrin, Vipin Villgran, Michael Nestasie, Feifan Chen, Brent Hardman, Khalid Malik

**Affiliations:** a Medicine Institute, Allegheny Health Network, 320 East North Avenue, Pittsburgh, PA, 15212, USA; b Division of Pulmonary and Critical Care, Allegheny Health Network, 320 East North Avenue, Pittsburgh, PA, 15212, USA; c Division of Pathology, Allegheny Health Network, 320 East North Avenue, Pittsburgh, PA, 15212, USA

**Keywords:** Lane Hamilton syndrome, Idiopathic membranous nephropathy, Celiac disease, Phosphoplipase A2 receptor

## Abstract

Lane Hamilton Syndrome is the rare association of idiopathic pulmonary hemosiderosis and Celiac Disease. The definitive pathophysiologic link is unknown, but the syndrome has been described as co-occurring along with other diseases. We describe the first reported case of Lane Hamilton Syndrome and idiopathic membranous nephropathy. We also hypothesize the possibility of an immune-mediated connection between the pathologies and propose a potential link of the phospholipase A2 receptor.

## 1 Introduction

Lane and Hamilton [1] first described the association of idiopathic pulmonary hemosiderosis (IPH) and Celiac Disease (CD) in 1971. Although they could not find a definitive pathophysiologic link between the two conditions, they hypothesized that abnormal iron metabolism might explain the association. Since their original case report, many concurrent IPH and CD cases have been described, and the presence of both diseases together has been coined Lane Hamilton Syndrome (LHS). A literature review of the infrequently reported LHS cases reveals incidences of other co-occurring pathologies, such as epilepsy and cerebral calcification syndrome [2], Retinitis Pigmentosa [3], chronic granulomatous disease [4], complete heart block [5], and cardiomyopathy [[6], [7], [8]]. While LHS has co-occurred with the diseases mentioned above, including autoimmune conditions, no published LHS case reports coincide with renal pathology. Furthermore, the disease is more common in the pediatric population, with most adult cases having a prolonged course, improved outcomes, and identification before the age of thirty [9].

Membranous nephropathy (MN) is the most common cause of nephrotic syndrome in nondiabetic adults. Historically, MN was divided into secondary MN when associated with an established cause such as systemic lupus erythematosus and idiopathic (primary) MN when no known cause was identified. However, understanding of primary MN has rapidly evolved in the last decade, and it is now known that the majority of cases of primary MN are associated with autoantibodies to the M-type phospholipase A2 receptor (PLA2R) [10]. To date, there have been four published cases of co-occurring CD and MN [[11], [12], [13], [14]]. We report the first case of MN, CD, and IPH, suggesting a possibly immune-linked process between LHS and MN.

## 2 Case presentation

The patient is a 39-year-old male with a past medical history of undifferentiated chronic pulmonary disease, chronic anemia, CD, atrial fibrillation, and PLA2R positive MN.

He reported that he had been healthy as a child and did not require regular medical care until his early twenties, when he began having recurrent episodes of symptomatic anemia characterized by severe fatigue receiving multiple blood transfusions. He reported receiving over thirty blood transfusions since his early twenties.

Five years before the current presentation, he underwent workup for celiac disease and was found to have elevated tissue transglutaminase IgA levels, compatible findings on small bowel biopsy, and presence of HLA-DQ2. He was not compliant with a celiac diet following his diagnosis but reported only mild gastrointestinal symptoms. Additionally, five years prior, he experienced respiratory failure with hemoptysis.

**Fig. 1:**
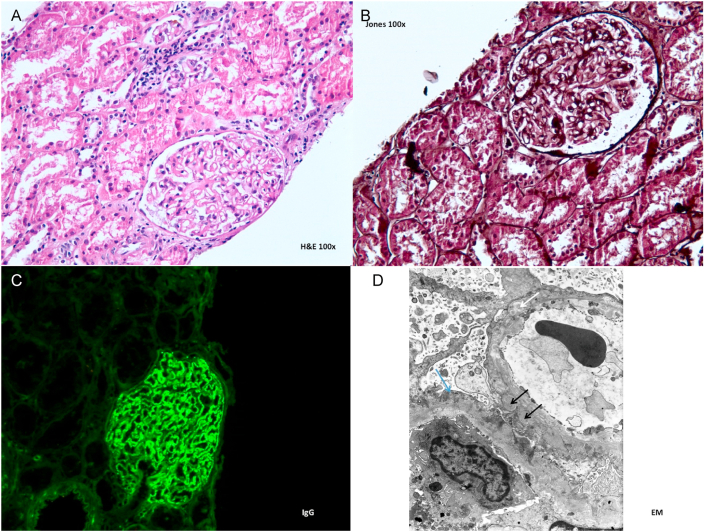
(A) Hematoxylin and eosin (H&E) stain shows the glomerulus which appears unremarkable under light microscopy. (B) The biopsy with Jones silver stain that is without spikes or pinhole vacuolations detected by light microscopy. (C) Immunofluorescence with global granular capillary loop IgG deposits (3+) (D) An electron microscopy picture of the glomerulus. The blue arrow demonstrates effacement of the foot processes or podocytes. The black arrow exhibits sub-epithelial amorphous deposits. The white arrow depicts a partially resorbed sub-epithelial deposit. (For interpretation of the references to colour in this figure legend, the reader is referred to the Web version of this article.)

Two years before presentation, he developed lower extremity edema and weight gain and was found to have nephrotic range proteinuria. Renal biopsy demonstrated membranous nephropathy (Fig. 1) with positive PLA2R staining performed at Arkana labs, and he was diagnosed with PLA2R associated MN, for which he was treated with angiotensin receptor blocker alone. Around this time, he was evaluated for dyspnea and abnormal chest imaging as well. He underwent bronchoscopy with transbronchial biopsy, which revealed evidence of alveolar hemosiderosis, endogenous pneumoconiosis, and phlebosclerosis. The patient did not recall specific medical treatment or diagnosis at that time.

The patient presented to our hospital after developing acute left-sided chest pain and dyspnea on exertion. He revealed a similar presentation a year earlier that resolved after drainage of a large left-sided pleural effusion. Since that admission, he had been doing well while adhering to a gluten-free diet.

On presentation, the patient was tachycardic, hypertensive, and hypoxic. His creatinine was elevated, and hemoglobin was stable. A CT angiogram chest revealed a moderate-sized right pleural effusion, emphysema, mediastinal adenopathy, and interstitial abnormalities. The patient was admitted for further evaluation by pulmonology and cardiothoracic surgery. A thoracentesis was performed on his right-sided pleural effusion, which was exudative based on Light's criteria. Infectious and rheumatologic workups were unrevealing, including ANA, ANCA, and anti-glomerular basement membrane antibodies. Drainage of the patient's pleural effusion significantly improved his presenting symptoms, and he was started on steroids for suspected LHS. Nephrology was consulted, and he was started on Cyclosporine for treatment of his MN.

**Fig. 2:**
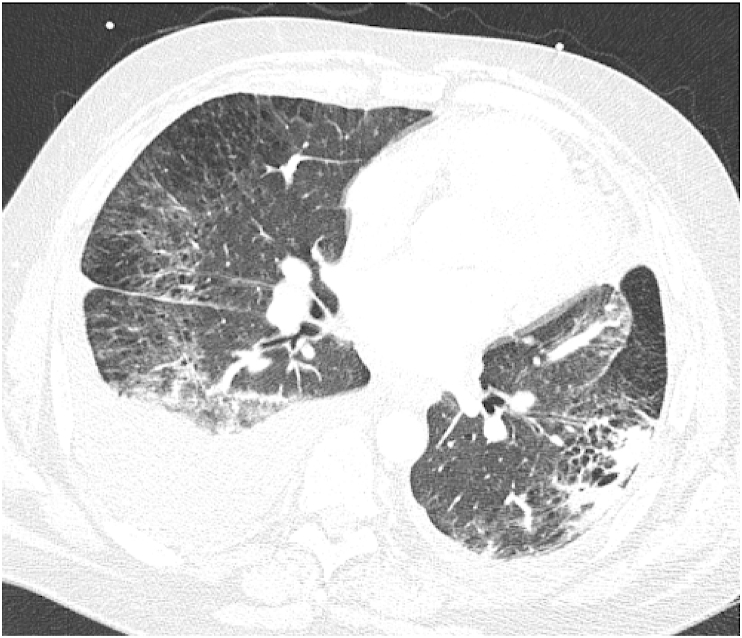
CT of the chest remarkable for a new right lower lobe consolidation and re-accumulation of right-sided pleural effusion, with a background of emphysema, and peripherally oriented ground-glass opacities with sub-pleural sparing in the lungs, likely related to fibrosis.

**Fig. 3:**
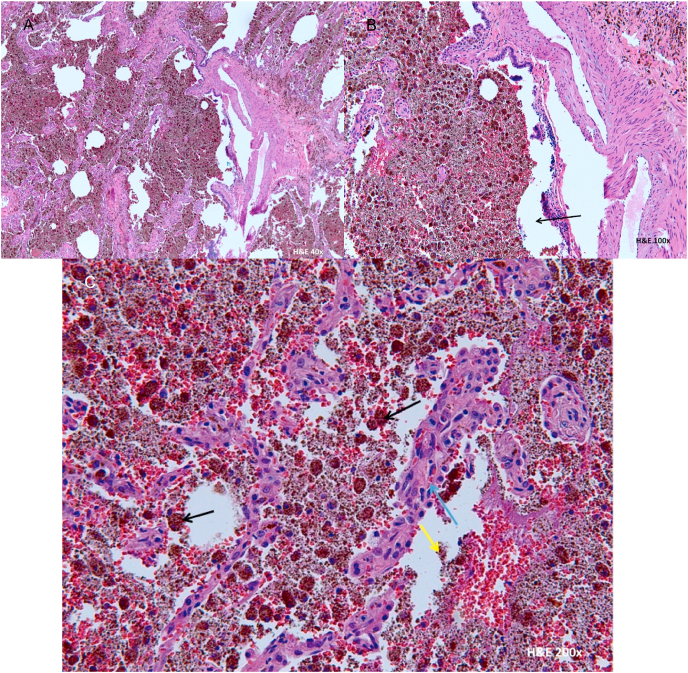
(A, B) Lung biopsy with red brown material filling up alveoli airspaces. (C) The blue arrow depicts type 2 pneumocytes, which line the alveoli, with hyperplasia. The yellow arrow demonstrates small red-brown granules of hemosiderin. The black arrow points out larger, denser red-brown macrophages which are phagocytosing the hemosiderin, called hemosiderin-laden macrophages. (For interpretation of the references to colour in this figure legend, the reader is referred to the Web version of this article.)

One week after discharge, he re-presented with dyspnea and a significant leukocytosis to 40.89 k/mcL. CT scan was notable for a new right lower lobe consolidation and re-accumulation of right-sided pleural effusion (Fig. 2). He completed a seven-day antibiotic course for pneumonia. Ultimately, a right-sided video-assisted thoracoscopic surgery (VATS) was performed to drain the pleural effusion and obtain wedge and peripheral lung biopsies. Pathology demonstrated benign lung parenchymal tissue with patchy areas of alveolar hemorrhage and an extensive collection of intra-alveolar hemosiderin containing macrophages consistent with pulmonary hemosiderosis (Fig. 3). Notably, during admission, iron and total iron-binding capacity were decreased at 14 mcg/dL and 242 mcg/dL, respectively, with ferritin elevated to 671 ng/mL.

The patient was eventually diagnosed with idiopathic pulmonary hemosiderosis after ruling out alternate causes of pulmonary hemosiderosis. His lung tissue was sent out to Arkana Laboratories and came back negative for PLA2R antibody. He was consequently discharged on corticosteroids and advised to adhere to a strict gluten-free diet.

## 3 Discussion

LHS is a rare disease with a poorly understood immunologic basis. While MN and CD are not necessarily rare, reported co-occurrence has been infrequent. We report the first case of a patient with both LHS and MN.

Initially, we hypothesized an association between LHS and MN might exist. Pulmonary renal syndromes may share common features due to similarities between the glomerular basement membrane and the alveolar basement membrane, such as type IV collagen, which is implicated in Goodpasture's syndrome. We considered the possible link of PLA2R expression on the alveolar basement membrane. This antibody is known to form immune complexes leading to podocyte damage within the kidney [15]; we considered that it might be attacking our patient's lungs as well. However, pathologic tissue staining was not consistent with this postulation, arguing against our hypothesis of PLA2R as the immune-mediated connection. Furthermore, immune complex deposition has not been shown in IPH. To our knowledge, there is no established protocol for staining lung tissue for PLA2R, making our negative test challenging to interpret with absolute certainty.

Notably, PLA2R positive staining does not definitively diagnose a primary MN based on a recent retrospective review that suggests in limited cases there may be a secondary cause such as malignancy (5%), a systemic autoimmune disease (5%), paraproteinemia (4%), viral or bacterial infections (0.4%), and non-steroidal anti-inflammatory use (1.8%) [16]. Our suspicion for a secondary PLA2R positive MN in our patient is low, given that he had none of the above. The possibility of hemosiderosis as the secondary cause of the patient's MN was considered [17]; however, we suspect this to be less likely. The patient was treated with cyclosporine and low dose prednisone, despite receiving multiple blood transfusions during this period he never required any phlebotomy treatments or iron chelators. Furthermore, hemosiderosis screening for patients who have received over twenty red blood cell transfusions is typically defined as ferritin levels ≥1000 ng/mL [18]. In reviewing the patients ferritin levels prior to the admission described in the case report, beginning in 2014, they are within normal limits, ranging from 32 to 179 ng/mL. However, should a similar case or question arise in the future, definitive renal biopsy staining with Perls' Prussian blue method could be considered.

While the patient may be unlucky to carry separate diagnoses of LHS and MN, we believe an association between CD and MN should still be considered, as there have been four reported cases of association. The first identified case occurred in Italy in a 32-year-old male diagnosed with MN, followed by diagnoses of CD and ulcerative colitis [11]. The second case described a 62-year-old male in the Netherlands with hypertension and anemia, diagnosed after duodenal and kidney biopsies [12]. The third case highlighted a 77-year-old male with hypertension, anemia, and acute renal failure diagnosed after a small bowel biopsy and a renal biopsy [13]. The fourth case was reported in a 35-year-old female who developed biopsy-proven CD after her first pregnancy and was found to have proteinuria, which led to a renal biopsy revealing MN [14]. Given that both CD and MN are immune-mediated, we suspect this rare but reported association is linked.

Furthermore, the missing link may provide insight into the relationship between IPH and CD, the LHS components. We suspect that our patient's concurrent MN and LHS is not an unfortunate coincidence but rather a single, connected autoimmune process, the basis of which has yet to be identified. While our case is the first report of concurrent LHS and MN, the association should be kept in mind given the likely, but to date undifferentiated, connection.

Given that LHS is believed to be immune-mediated, initial treatment with steroids is recommended in order to prevent recurrent episodes of diffuse alveolar hemorrhage in the setting of IPH. Successful treatment of IPH with a combination of oral steroids and azathioprine or hydroxychloroquine has also been reported [19]. Maintaining a gluten-free diet has been shown to improve symptoms of both CD and IPH in the setting of LHS [20]. Recognition and treatment of LHS are crucial to prevent the disease's natural progression, which can result in worsening pulmonary hemosiderosis and fibrosis. Lung transplantation has been unsuccessful in patients with a history of IPH, as recurrence of bleeding has been reported [21].

## Funding

This research did not receive any specific grant from funding agencies in the public, commercial, or not-for-profit sectors.

## Consent for publication

Written informed consent for publication was obtained from the patient.

## Declaration of competing interest

None.
